# Supplementary material for: Prevalence and characteristics of cannabis-induced toxicoses in pets: Results from a survey of veterinarians in North America
Source: PLoS One. 2022 Apr 20;17(4):e0261909. doi: 10.1371/journal.pone.0261909 (PMC9020701; doi:10.1371/journal.pone.0261909)
Supplement: S1 File — (PDF) [file pone.0261909.s001.pdf]

Supplementary Data: Survey questions

CONSENT (French version below English text)

You are invited to participate in a web-based online survey on Cannabis Toxicosis In Pets. This is a research project being conducted by Richard Quansah Amissah, a postdoctoral fellow in Dr. Jibran Khokhar's laboratory in the Department of Biomedical Sciences, Ontario Veterinary College, University of Guelph at the University of Guelph. It should take between 5 to 10 minutes to complete.

To be eligible to participate in this survey, you must:

1. be a practicing veterinarian,
2. be working in either Canada or the USA,
3. be treating pets, and
4. have treated or been presented with cases involving cannabis toxicosis in pets.

Your participation in this survey is voluntary. You may refuse to participate in the research or exit the survey at any time. You are free to decline to answer any particular question you do not wish to answer for any reason. You will receive no direct benefits from participating in this research study. However, your responses may help us learn more about and understand cannabis toxicosis in pets and may contribute to the development of better therapies for its treatment. There are no foreseeable risks involved in participating in this study other than those encountered in day-to-day life. Your survey answers will be sent to a link at Qualtrics.com where data will be stored in a password protected electronic format. Qualtrics does not collect identifying information such as your name, email address, or IP address. Therefore, your responses will remain anonymous meaning that no one will be able to identify you or your answers, and no one will know whether or not you participated in the study. If you have questions at any time about the study or the procedures, you may contact my research supervisor, Dr. Jibran Khokhar via email at jkhokhar@uoguelph.ca.

Please select your choice below. Clicking on the “Agree” button indicates that

You have read the above information

You voluntarily agree to participate

Please click "Agree" to complete the questionnaire or "Disagree" to opt out of the survey.

Vous êtes invités à participer un sondage en ligne sur la toxicose du cannabis chez les animaux de compagnie. Il s'agit d'un projet de recherche mené par Richard Quansah Amissah, stagiaire postdoctoral au

laboratoire de Dr. Jibran Khokhar, département des sciences biomédicales, Ontario Veterinary College, à l'Université de Guelph. Le sondage devrait prendre entre 5 et 10 minutes à compléter.

Pour être éligible à participer à ce sondage, vous devez:

1. Être un vétérinaire en exercice,
2. Travailler au Canada ou aux États-Unis,
3. Traiter les animaux de compagnie, et
4. Avoir traité ou avoir vu des cas de toxicose au cannabis chez des animaux de compagnie.

Votre participation à cette enquête est volontaire. Vous pouvez refuser de participer à la recherche ou quitter l'enquête à tout moment. Vous êtes libre de refuser de répondre à toute question particulière à laquelle vous ne souhaitez pas répondre pour quelque raison que ce soit. Vous ne recevrez aucun avantage direct en participant à cette étude de recherche. Cependant, vos réponses peuvent nous aider à en savoir plus et à comprendre la toxicose au cannabis chez les animaux de compagnie et peuvent contribuer au développement de meilleures thérapies pour son traitement. Il n'y a pas de risques prévisibles liés à la participation à cette étude autres que ceux rencontrés dans la vie de tous les jours. Vos réponses au sondage seront envoyées à un lien sur Qualtrics.com où les données seront stockées dans un format électronique protégé par mot de passe. Qualtrics ne collecte pas d'informations d'identification telles que votre nom, votre adresse courriel ou votre adresse IP. Par conséquent, vos réponses resteront anonymes, ce qui signifie que personne ne pourra vous identifier ou identifier vos réponses, et personne ne saura si vous avez participé ou non à l'étude. Si vous avez des questions à tout moment sur l'étude ou les procédures, vous pouvez contacter mon directeur de recherche, le Dr Jibran Khokhar par courriel à jkhokhar@uoguelph.ca.

Veuillez sélectionner votre choix ci-dessous. Cliquer sur le bouton « Accepter » indique que

Vous avez lu les informations ci-dessus

Vous acceptez volontairement de participer

Veuillez cliquer sur « J'accepte » pour remplir le questionnaire ou « Je ne suis pas d'accord » pour vous retirer de l'enquête.

○ Agree (J'accepte)

○ Disagree (Je ne suis pas d'accord)

End of Block: CONSENT

Start of Block: SCREENER

Q0 Before proceeding further, do you answer "Yes" to all the following questions:

Are you a practicing veterinarian?
Do you work either in Canada or the USA?
Do you treat pets?
Have you been presented with cases involving "suspected or confirmed" cannabis toxicosis in pets?

○ Yes

○ No

End of Block: SCREENER

Start of Block: DEMOGRAPHIC INFORMATION

Q1
In which country do you practice?

○ Canada

○ US

Q1a In which province do you practice?

▼ Alberta ... Yukon

Q1b In which state do you practice?

▼ Alabama ... Wyoming

*

Q2 In which city do you practice?

____

Q3 In which setting is the hospital/clinic where you work?

- ○ Urban
- ○ Sub-urban
- ○ Rural

Q4 Which type of veterinary medicine do you practice?

- ○ Emergency Medicine
- ○ General Medicine
- ○ Other. Please specify. ____

End of Block: DEMOGRAPHIC INFORMATION

Start of Block: EXPERIENCE AS A VETERINARIAN

Q5 How many pets do you personally see/treat in a month in general?

- ○ 0 – 50
- ○ 51 – 100
- ○ 101 – 150
- ○ 151 – 200
- ○ > 200

Q6 Which classes of pets do you treat? Please select all that apply.

- ☐ Mammals
- ☐ Reptiles
- ☐ Invertebrates
- ☐ Birds
- ☐ Amphibians
- ☐ Fish

Q6a Which types of mammals do you treat? Please select all that apply.

- ☐ Cats
- ☐ Dogs
- ☐ Horses
- ☐ Pigs
- ☐ Goats
- ☐ Sheep
- ☐ Others. Please specify. ____

Q6b Which types of reptiles do you treat? Please select all that apply.

- ☐ Snakes
- ☐ Lizards
- ☐ Iguanas
- ☐ Tortoises
- ☐ Turtles
- ☐ Others. Please specify. ____

Q6c Which types of invertebrates do you treat? Please select all that apply.

- ☐ Tarantulas
- ☐ Scorpions
- ☐ Insects
- ☐ Millipedes
- ☐ Ants
- ☐ Others. Please specify. ____

Q6d Which types of birds do you treat? Please select all that apply.

- ☐ Doves
- ☐ Canaries
- ☐ Parrots
- ☐ Finches
- ☐ Cockatoos
- ☐ Others. Please specify. ____

Q6e Which types of amphibians do you treat? Please select all that apply.

- ☐ Frogs
- ☐ Axolotls
- ☐ Toads
- ☐ Salamanders
- ☐ Newts
- ☐ Others. Please specify. ____

Q6f Which types of fish do you treat? Please select all that apply.

- ☐ Beta
- ☐ Goldfish
- ☐ Angelfish
- ☐ Catfish
- ☐ Guppies
- ☐ Others. Please specify. ____

End of Block: EXPERIENCE AS A VETERINARIAN

Start of Block: EXPERIENCE WITH TOXICOSIS

Q7 How was/were the diagnosis/diagnoses reached for most of the "suspected or confirmed" cannabis toxicosis cases? Please select all that apply.

- ☐ Based on supportive clinical signs
- ☐ History of possible exposure
- ☐ History of known exposure
- ☐ Use of OTC Urine Drug Tests with a positive THC test result
- ☐ Other. Please specify. ____

Q8 How many "suspected or confirmed" cannabis toxicosis cases were you presented with **yearly** as the primary veterinarian **before cannabis legalization in Canada** (October 17, 2018)?

- 1 – 12
- 13 – 24
- 25 – 36
- 37 – 48
- 49 – 60
- > 60

Q9 How many "suspected or confirmed" cannabis toxicosis cases are you presented with **yearly** as the primary veterinarian **following cannabis legalization in Canada** (October 17, 2018)?

- 1 – 12
- 13 – 24
- 25 – 36
- 37 – 48
- 49 – 60
- > 60

Q10 Please select all the pet species that presented with "suspected or confirmed" cannabis toxicosis.

- [ ] Cats
- [ ] Dogs
- [ ] Horses
- [ ] Pigs
- [ ] Goats
- [ ] Sheep
- [ ] Snakes
- [ ] Lizards
- [ ] Iguanas
- [ ] Tortoises
- [ ] Turtles
- [ ] Doves
- [ ] Canaries
- [ ] Parrots
- [ ] Finches
- [ ] Cockatoos

- [ ] Frogs
- [ ] Axolotls
- [ ] Toads
- [ ] Salamanders
- [ ] Newts
- [ ] Betas
- [ ] Goldfish
- [ ] Angelfish
- [ ] Catfish
- [ ] Guppies
- [ ] Others. Please specify. ____

Q11 Among the pet species selected above, which species frequently presented with "suspected or confirmed" cannabis toxicosis? Please select all that apply.

- ☐ Cats
- ☐ Dogs
- ☐ Horses
- ☐ Pigs
- ☐ Goats
- ☐ Sheep
- ☐ Snakes
- ☐ Lizards
- ☐ Iguanas
- ☐ Tortoises
- ☐ Turtles
- ☐ Doves
- ☐ Canaries
- ☐ Parrots
- ☐ Finches
- ☐ Cockatoos

- [ ] Frogs
- [ ] Axolotls
- [ ] Toads
- [ ] Salamanders
- [ ] Newts
- [ ] Betas
- [ ] Goldfish
- [ ] Angelfish
- [ ] Catfish
- [ ] Guppies
- [ ] Others. Please specify. ____

End of Block: EXPERIENCE WITH TOXICOSIS

Start of Block: CAUSES OF TOXICOSIS

Q12 In most cases, were the causes of the "suspected or confirmed" cannabis toxicosis known?

- ○ Yes
- ○ No

Q13 What product(s) often led to "suspected or confirmed" cannabis toxicosis in the pets you treated? Please select all that apply.

- ☐ Edibles
- ☐ Capsulated cannabis products
- ☐ Dried cannabis
- ☐ Fresh/green cannabis plant material
- ☐ Tablets containing cannabinoids
- ☐ Cannabis smoke/vapor
- ☐ Cannabis topicals
- ☐ Cannabis concentrates
- ☐ Others. Please specify. ____

Q14 How were the products that led to the "suspected or confirmed" cannabis toxicosis in the pets obtained?

- ☐ From government regulated producers
- ☐ From home cultivated plants
- ☐ From the black market
- ☐ Unknown
- ☐ Others. Please specify. ____

Q15 What was/were the most popular route(s) of exposure to the cannabis products for pets that presented with "suspected or confirmed" cannabis toxicosis? Please select all that apply?

- ▢ Ingestion
- ▢ Inhalation
- ▢ Topical application
- ▢ Tinctures
- ▢ Others. Please specify. ____

Q16 What was/were the most common suspected reason(s) for exposure?

- ▢ Intentional administration for treatment
- ▢ Intentional administration for recreation
- ▢ Ingestion while unattended
- ▢ Unknown
- ▢ Other. Please specify. ____

End of Block: CAUSES OF TOXICOSIS

Start of Block: SYMPTOMS OF TOXICOSIS

Q17 Which symptoms were frequently present in the pets following "suspected or confirmed" cannabis toxicosis? Please select all that apply.

- ☐ Anorexia
- ☐ Bradycardia
- ☐ Disorientation
- ☐ Diarrhea
- ☐ Hypertension
- ☐ Hypotension
- ☐ Increased anxiety
- ☐ Dry mouth/excessive drinking
- ☐ Polyphagia
- ☐ Vocalizing/crying
- ☐ Seizures
- ☐ Tachycardia
- ☐ Vomiting
- ☐ Lateral recumbency
- ☐ Urinary incontinence
- ☐ Lethargy

☐ Stupor/obtundation

☐ Hypothermia

☐ Tremors

☐ Agitation

☐ Respiratory depression

☐ Ataxia

☐ Mydriasis

☐ Hyperesthesia

☐ Twitching

☐ Ptyalism

☐ Others. Please specify ____

End of Block: SYMPTOMS OF TOXICOSIS

Start of Block: LOOP AND MERGE 2

Q18 Among the frequencies below, please select the one that accurately describes the frequency of ${lm://Field/1}.

	1	2	3	4	
Very often	○	○	○	○	Rare

End of Block: LOOP AND MERGE 2

Start of Block: SYMPTOM SEVERITY

Q19 Were any of the symptoms severe?

- ○ Yes
- ○ No

Q20 Which symptoms were severe? Please select all that apply.

- ☐ Anorexia
- ☐ Bradycardia
- ☐ Disorientation
- ☐ Diarrhea
- ☐ Hypertension
- ☐ Hypotension
- ☐ Increased anxiety
- ☐ Dry mouth/excessive drinking
- ☐ Polyphagia
- ☐ Vocalizing/crying
- ☐ Seizures
- ☐ Tachycardia
- ☐ Vomiting
- ☐ Lateral recumbency
- ☐ Urinary incontinence
- ☐ Lethargy

- [ ] Stupor/obtundation
- [ ] Hypothermia
- [ ] Tremors
- [ ] Agitation
- [ ] Respiratory depression
- [ ] Ataxia
- [ ] Mydriasis
- [ ] Hyperesthesia
- [ ] Twitching
- [ ] Ptyalism
- [ ] Other. Please specify. ____

End of Block: SYMPTOM SEVERITY

Start of Block: FOLLOW UP

Q21 How long was the treatment/monitoring course for most of the "suspected or confirmed" cannabis toxicosis cases?

- Treated as out-patient
- Hospitalized for
- Hospitalized for 6-12 hours
- Hospitalized for 12-24 hours
- Hospitalized for 24-48 hours
- Hospitalized for 48-72 hours
- Hospitalized for >72 hours

Q22 Did any of the pets that presented with "suspected or confirmed" cannabis toxicosis return for follow-up?

- Yes
- No

Q23 How often did the pets that presented with "suspected or confirmed" cannabis toxicosis return for follow-up?

- Always
- Often
- Sometimes
- Rarely
- Never

Q24 Were any residual symptoms present?

- ◯ Yes
- ◯ No

Q25 What were the residual symptoms? Please select all that apply.

- ☐ Anorexia
- ☐ Bradycardia
- ☐ Disorientation
- ☐ Diarrhea
- ☐ Hypertension
- ☐ Hypotension
- ☐ Increased anxiety
- ☐ Dry mouth/excessive drinking
- ☐ Polyphagia
- ☐ Vocalizing/crying
- ☐ Seizures
- ☐ Tachycardia
- ☐ Vomiting
- ☐ Lateral recumbency
- ☐ Urinary incontinence
- ☐ Lethargy

☐ Stupor/obtundation

☐ Hypothermia

☐ Tremors

☐ Agitation

☐ Respiratory depression

☐ Ataxia

☐ Mydriasis

☐ Hyperesthesia

☐ Twitching

☐ Ptyalism

☐ Other. Please specify. ____

End of Block: FOLLOW UP

Start of Block: TREATMENT

Q26 Did the symptoms completely resolve for most cases following appropriate veterinary care?

○ Yes

○ No

Q27 What type(s) of care was/were often provided for "suspected or confirmed" cannabis toxicosis?

- ☐ Outpatient monitoring and supportive care
- ☐ In-hospital monitoring only
- ☐ IV fluids
- ☐ Induction of emesis
- ☐ Administration of activated charcoal
- ☐ Administration of anti-emetics
- ☐ Thermal support (warming/cooling)
- ☐ EKG monitoring
- ☐ BP monitoring
- ☐ Use of anti-arrhythmics
- ☐ Use of anti-convulsants
- ☐ Administration of IV intra-lipids
- ☐ Other. Please specify. ____

Q28 What is the average cost of the cannabis-related toxicosis visits?

- $ 1-500
- $ 501 – 1000
- $ 1001 – 1500
- $ 1501 – 2000
- > $ 2000

End of Block: TREATMENT

Start of Block: DEATH FROM TOXICOSIS

Q29 Did any of the cases die due to cannabis toxicosis?

- Yes
- No

---

*

Q30 How many of the cases died due to cannabis toxicosis? Please enter a numerical figure.

____

Q31 What was/were the cause(s) of death(s)? Please select all that apply.

- ☐ Cardiac arrhythmia
- ☐ Aspiration pneumonia
- ☐ Trauma from fall due to ataxia
- ☐ Uncontrolled seizures
- ☐ Euthanasia
- ☐ Other. Please specify. ____

End of Block: DEATH FROM TOXICOSIS
